# Acute respiratory distress syndrome (ARDS) after pressurized intraperitoneal aerosol chemotherapy with oxaliplatin: a case report

**DOI:** 10.1515/pp-2021-0126

**Published:** 2021-10-05

**Authors:** Emilie Thibaudeau, Corinne Brianchon, Jean-Luc Raoul, Frédéric Dumont

**Affiliations:** Department of Oncological Surgery, Institut de Cancérologie de l’Ouest, Saint-Herblain, France; Department of Anesthesia, Institut de Cancérologie de l’Ouest, Saint-Herblain, France; Department of Medical Oncology, Institut de Cancérologie de l’Ouest, Saint-Herblain, France

**Keywords:** acute respiratory distress syndrome, complications, oxaliplatin, peritoneal carcinomatosis, PIPAC

## Abstract

Pressurized intraperitoneal aerosol chemotherapy (PIPAC) is a new drug delivery method for intraabdominal cavity chemotherapy. It combines the benefits of a minimally invasive approach (low morbidity and easy to repeat) with the pharmacokinetic advantages of intraperitoneal administration and tolerance seems excellent. We would like to report one case of a serious adverse event, acute respiratory distress syndrome, which is likely related to oxaliplatin administration; all signs disappeared within a few days.

## Introduction

Despite significant improvements in the systemic treatment of metastatic colorectal cancer, peritoneal metastatic spreads have a dismal prognosis [1], partly because of poor vascularization of the peritoneum. Locoregional chemotherapy aims to increase intratumoral concentrations of the chemotherapeutic agent. Pressurized intraperitoneal aerosol chemotherapy (PIPAC) is a new drug delivery method that applies chemotherapy into the abdominal cavity as an aerosol under pressure (CO_2_) during laparoscopy procedures. It combines the benefits of a minimally invasive approach with low morbidity, is easy to repeat and has the pharmacokinetic advantages of intraperitoneal administration. Moreover, high-pressure laparoscopy enhances tumor penetration of cytotoxic drugs [2] and aerosol delivery allows good spatial diffusion of the chemotherapy [3].

Many series have highlighted the good tolerance of PIPAC: postoperative mortality is less than 1% with incidences of grade three and four serious adverse events of 17–23% and 0–3%, respectively [4].

Here we report the first case of acute respiratory distress syndrome (ARDS) after a PIPAC session, likely related to oxaliplatin.

## Case presentation

A 60-year-old nonsmoking woman with no past medical history was treated for pT4bN2b(20/34)M1c (peritoneal carcinomatosis) colonic adenocarcinoma from the left side with 12 cycles of a triple chemotherapy regimen (FOLFIRINOX). Tolerance was excellent with no allergy or respiratory side effects.

Because of unresectable carcinomatosis (initial peritoneal carcinomatosis index (PCI) at 27), she received PIPAC with oxaliplatin (90 mg/m^2^). She was ECOG Performance Status one and her BMI was 27. During the first PIPAC session, PCI was evaluated at 27 and the surgical procedure was straightforward. Hours after the procedure, however, she presented with severe abdominal pain for a few days, requiring opioids. During the second PIPAC session in October 2020, the peritoneum was highly inflamed, a sign of chemical peritonitis [5] (Supplementary Video 1, showing the peritoneal appearance during the first and second sessions) likely explaining the postoperative pain. Such chemical peritonitis may be responsible for severe peritoneal sclerosis (SPS) associated with a systemic inflammatory response and for blood-stained ascites [6].

**Supplementary Video 1:** 

Two hours after extubation, she presented with sudden respiratory failure with tachypnea (between 25 and 40 breaths per min), hypoxemia (oxygen saturation (SaO_2_) 88%) and tachycardia (120 beats per min) requiring 8 L/min of oxygen therapy. Arterial blood gas showed pH 7.37, partial pressure of carbon dioxide (PaCO_2_) 37 mmHg, partial pressure of oxygen (PaO_2_) 67 mmHg, SaO2 92.7%, lactate 2.3 mmol/L, HCO_3−_ 19.8 mmol/L, and total CO_2_ 21 mmol/L.

**Figure 1:**
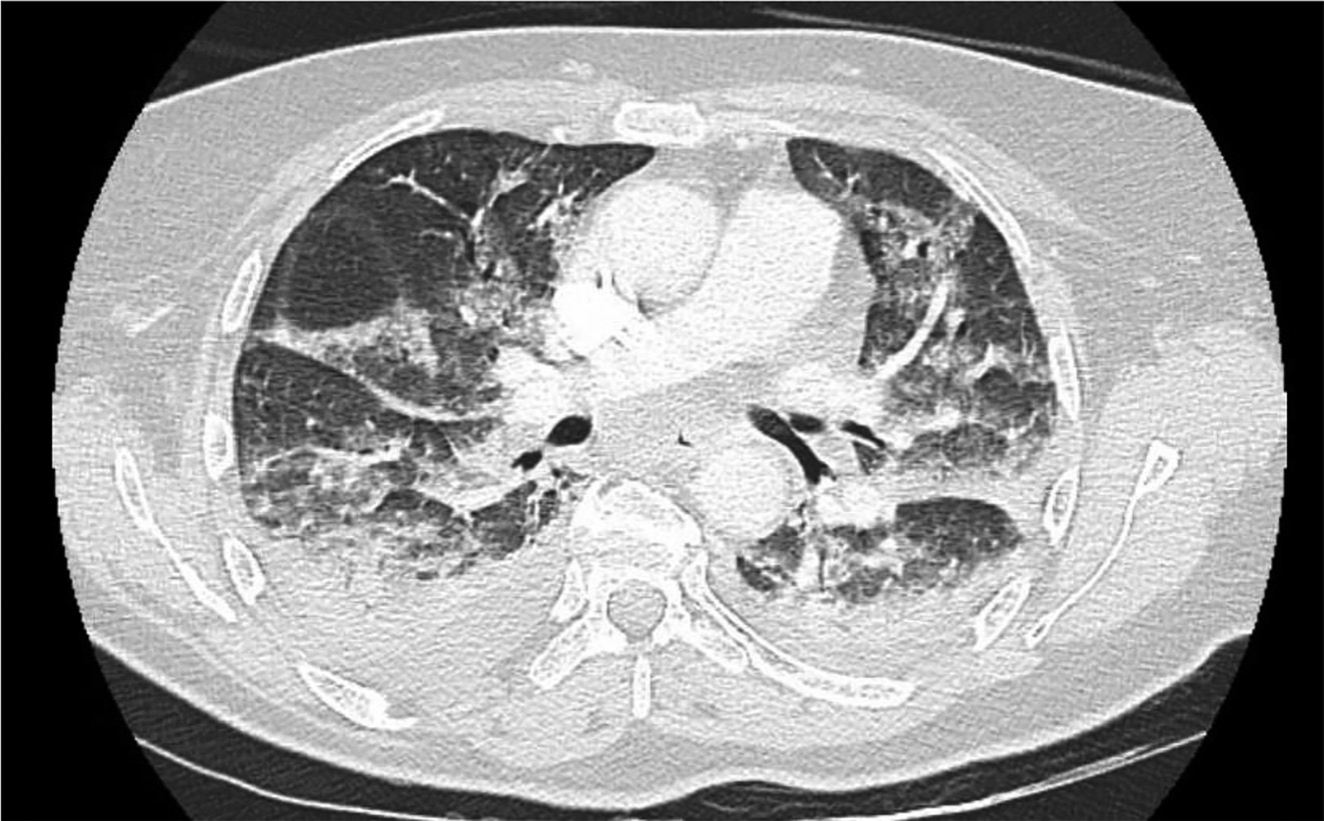
CT pulmonary angiography and thoracic–abdominal–pelvic computed tomography during respiratory failure with no pulmonary embolism, no sign of post PIPAC complications in the abdomen but bilateral pleural diffusion with passive atelectasis and alveolar–interstitial syndrome.

An enhanced thoracoabdominal computed tomography (CT) scan was normal, eliminating a severe pulmonary embolism and intraabdominal complication of the surgical procedure. On thoracic slices, however, there was low-abundance bilateral pleural effusion, with passive atelectasis and a bilateral alveolar-interstitial syndrome not found on the pretreatment CT scan (Figure 1).

According to the Berlin criteria [7], the diagnosis of post PIPAC moderate ARDS was retained: the patient presented with impaired oxygenation with PaO_2_/FiO_2_ = 111 mmHg (PaO_2_ = 73 mmHg and FiO_2_ = 60%) and positive end-expiratory pressure (PEEP) = 5 cmH_2_O.

She was transferred to an intensive care unit and received non-invasive ventilation (NIV) with PEEP 5 cmH_2_O and HELP 4 cmH_2_O for 1 h three times daily and corticosteroid therapy 60 mg/day for 3 days.

Her clinical condition improved, the NIV was stopped after three days and replaced with 2 L/min oxygen therapy; she was weaned off oxygen therapy five days after the ARDS and then discharged. No further PIPAC was proposed.

## Discussion

In our case, the ARDS seems related to PIPAC with oxaliplatin because all other etiologies were ruled out. Anesthesia was not suspected because this period was uneventful; the patient did not present with bronchospasm at induction and was given lung protective ventilation 6–8 mL/kg with positive end-expiratory pressure (PEEP). Infection was rejected because there was no inhalation or bronchial superinfection during anesthesia: she was and remained afebrile; biological parameters were normal as were bacteriological examinations; polymerase chain reaction for coronavirus disease was negative; cytobacteriological examination of sputum was also negative. Cardiac origin was unlikely: no cardiac history, no heart failure; despite tachycardia (120 beats/min), blood pressure remained normal; troponin and the N-terminal fragment of the brain natriuretic peptide precursor were normal. There was no intraabdominal complication or pulmonary embolism on the CT scan.

The first diagnosis that can be established here is an ARDS caused by chemical peritonitis. Demtroder [5] has shown that PIPAC with oxaliplatin can induce postoperative inflammatory syndrome (with significant increases in C-reactive protein) with abdominal pain and fever, symptoms corresponding to chemical peritonitis. Those results were confirmed in 2018 [8]. It seems that oxaliplatin induces a more serious inflammatory syndrome than the combination of cisplatin and doxorubicin. The inflammatory syndrome does not depend on the number of PIPAC sessions given to each patient but is correlated positively to the tumor load.

This chemical peritonitis leading to SPS associated with a systemic inflammatory response may be responsible for blood-stained ascites [6]. In our case we have many arguments for significant chemical peritonitis after the first PIPAC. The patient had serious peritoneal carcinomatosis (PCI at 27/39) and developed severe abdominal pain requiring opioids after the first PIPAC session. During the second session, the coelioscopy revealed white plaque-like material at the surface of the peritoneum, the first signs of peritoneal sclerosis caused by chemical peritonitis. Although such chemical peritonitis is quite frequent, only a few cases of ARDS have been previously reported following PIPAC; but one can hypothesize that this severe inflammatory process may trigger and emphasize oxaliplatin-related toxicity.

The second explanation for this ARDS is rare oxaliplatin toxicity. During PIPAC using oxaliplatin, systemic absorption is important and expected to provide systemic efficacy [9]. In animal models, the maximum blood concentration was observed 1 h after the experiment’s onset [10]. Such systemic exposure to oxaliplatin after (electrostatic) PIPAC is comparable to that observed after systemic chemotherapy [11]. Besides frequent oxaliplatin toxicities (peripheral neuropathy, nausea, vomiting and bone marrow suppression), there can be unusual hypersensitivity reactions and pulmonary toxicity which is sometimes fatal, and some cases have been reported after PIPAC with oxaliplatin or cisplatin [12].

An extensive review of the English-language literature on oxaliplatin lung toxicities found over 40 cases of pulmonary toxicity directly related to oxaliplatin [13], [14], [15]. There is a male preponderance (male 75%) and mean age of 66.7 years (male 68.2 and female 62.3). Clinical presentation is heterogenous. Symptoms range from mild to severe and evolution varies from complete resolution to death [16]. Histologic findings are also diverse: organizing pneumonia, diffuse alveolar damage or nonspecific interstitial pneumonia. For De Weerdt [15], this complication arises after a median of eight cycles of FOLFOX (range 1–22) and a mean cumulative dose of 729.8 mg/m^2^ (our patient received oxaliplatin 14 times, giving a total dose of 2,010 mg/m^2^). Patients who needed intubation had a worse prognosis with very high mortality of 76.9%.

In our phase 1 study [9, 17], one of the dose-limiting toxicities was an allergic reaction to oxaliplatin in a patient previously treated with oxaliplatin. Hypersensitivity reactions to oxaliplatin are frequent (10–20% of patients) and usually occur after cycle 6, with severe reactions mainly observed after 8–10 sessions [18].

Therefore, in the authors’ opinion, PIPAC should be introduced earlier in the course of patients’ chemotherapy treatment so that it is less likely to result in toxicity related to the total cumulative dose of oxaliplatin.

The intensity of the chemical peritonitis may be an additional factor concurring to this toxicity, either by the release of toxic components or increased systemic absorption of oxaliplatin. Moreover in our PIPOX phase I–II dose-escalation study of oxaliplatin, the pharmacokinetic research shows that the level of systemic oxaliplatin was not modified by the number of PIPAC sessions given or whether or not there is an inflammatory aspect of the peritoneum.

In conclusion, this first reported case of ARDS following PIPAC with oxaliplatin may be related to a hypersensitivity reaction to oxaliplatin or to chemical peritonitis. It appears impossible to repeat PIPAC with oxaliplatin or with another platinum derivative (cross-reactivity to other platinum salts). To reduce the risks of oxaliplatin hypersensitivity, it could be of interest to use PIPAC with oxaliplatin earlier.
